# Low and No-Contact Euthanasia: Associated Ethical Challenges Experienced by Veterinary Team Members during the Early Months of the COVID-19 Pandemic

**DOI:** 10.3390/ani12050560

**Published:** 2022-02-23

**Authors:** Anne Quain, Siobhan Mullan, Michael P. Ward

**Affiliations:** 1 Sydney School of Veterinary Science, University of Sydney, Sydney, NSW 2006, Australia; michael.ward@sydney.edu.au; 2 School of Veterinary Medicine, University College Dublin, D04 V1W8 Dublin, Ireland; siobhan.mullan@ucd.ie

**Keywords:** euthanasia, pandemic, COVID-19, SARS-CoV-2, physical distancing, human-animal bond, ethics, moral distress, fear-free, low stress, PPE

## Abstract

**Simple Summary:**

During the COVID-19 pandemic, many veterinary practices have been required to move to a low or no-contact consultation model to minimise the risk of SARS-CoV-2. Utilising data from a global survey, we explored the experiences of veterinary team members performing low and no-contact euthanasia during the early months of the COVID-19 pandemic. We found that low and no-contact euthanasia were encountered as common and/or stressful ethical challenges in the pandemic. In order to minimise the potential negative impacts of low and no-contact euthanasia on veterinary team members, clients and animal patients, there is a need for a toolkit of protocols to assist veterinary team members in provision of low-contact euthanasia, and avoidance of no-contact euthanasia wherever possible.

**Abstract:**

Background: During the ongoing COVID-19 pandemic, many veterinary practices around the world have shifted to a low or no-contact consultation model to ensure the safety of their team members and clients, and comply with public health orders, while continuing to provide veterinary care. Methods: We performed reflexive thematic analysis on a subset of data collected using a mixed-methods survey of veterinary team members globally. Results: There were 540 valid responses available for analysis. Low and no-contact euthanasia we raised as a common and/or stressful ethical challenge for 22.8% of respondents. We identified five key themes: no-contact euthanasia as a unique ethical challenge; balancing veterinary team safety with the emotional needs of clients; low and no-contact protocols may cause or exacerbate fear, anxiety and distress in veterinary patients; physical distancing was more challenging during euthanasia consultations; and biosecurity measures complicated communication around euthanasia and end-of-life decision making. Recommendations: In light of concerns highlighted by respondents, we recommend the development of a toolkit of protocols that will assist veterinary team members in performing low-contact euthanasia in a range of circumstances, in alignment with their values and professional ethical codes. Professional bodies may be involved in developing, updating and disseminating this information, and ensuring a continuous supply chain of PPE.

## 1. Introduction

The World Health Organisation (WHO) declared a global pandemic on 11 March 2020 [1]. The COVID-19 pandemic led to major changes in veterinary practice to minimise the risk of transmission of SARS-CoV-2 in veterinary settings and in some instances comply with public health orders, including the increased use of personal protective equipment (PPE), and low and no-contact consultations to facilitate physical distancing.

For the purposes of this discussion, “low-contact” refers to strategies aimed at minimising physical contact with clients, such as minimising the number of clients in the consultation room, physical distancing, and requesting that clients wear PPE during the consultation. “No-contact” refers to strategies aimed at eliminating physical contact with clients. In such instances, clients may have had verbal contact with veterinary team members, for example via telephone or internet, to communicate their concerns, provide a patient history or give consent, but were required to remain outside of the premises at all times, for example in the case of “drop-off” or “curbside” consultations, or telemedicine.

Due to a surge in demand, disruption of global supply chains, shortage of raw materials for production, competition between countries for PPE and in some cases interception of PPE imports, there was a global shortage of PPE [2]. In particular, masks, goggles, face shields, gowns and N95 respirators were in very short supply [3]. Shortages of PPE in human healthcare settings left those caring for patients with COVID-19 extremely vulnerable to COVID-19-associated morbidity and mortality [4,5]. Healthcare workers were also deemed an important source of SARS-CoV-2 transmission [5]. These factors made protection of healthcare workers from infection a priority for disease control. Vaccinations were not approved for use until December 2020 in the UK, and in many countries not until much later [6,7]. With no approved vaccinations or effective treatment, physical distancing, PPE, and minimising the duration of proximity to others where physical distancing was not possible, were key elements of prevention.

For this reason, veterinary professional organisations and associations such as the American Veterinary Medical Association, promoted conserving PPE by postponing elective procedures, extending the use of disposable PPE or even reusing disposable PPE in some circumstances [8].

To minimise the risk of transmission of SARS-CoV-2 between clients and veterinary team members in veterinary clinical settings, most amended their practice to align with local public health orders or recommendations; to limit service provision only to “essential” services; to reduce the number of veterinary team members on site at any one time, and minimise client contact by limiting the number of clients entering veterinary premises, or to exclude clients from veterinary premises entirely [9,10,11]. 

In 2020 we surveyed veterinary team members around the world about the types of ethically challenging situations they had faced since the beginning of the pandemic. The results are published elsewhere [12,13]. We identified “no-contact consultations” in general, particularly “no-contact euthanasia consultations”, as distinct, novel types of ethically challenging situations faced by veterinary team members. 

Euthanasia is commonly performed in veterinary contexts [14,15,16,17,18,19,20]. Derived from the Greek “eu” for good and “thanatos”, pertaining to death, “euthanasia” describes the killing of an animal in such a way that minimises pain and distress to the animal patient, and emotional distress of those present, including animal owners [19]. While historically it was commonplace to separate animals from clients at the time of euthanasia, best practice is now keeping bonded humans and animals together, or at least providing that option for clients [21].

Previous surveys have identified euthanasia of animals as a source of moral stress and moral distress for veterinarians in particular [22,23,24,25]. Whether veterinary team members experience euthanasia as an ethical challenge may depend on the indication or reasons for a euthanasia request [26]. In preventing veterinary team members from keeping bonded humans and animals together, low and no-contact euthanasia may violate their expectations/values/beliefs around what constitutes a good death. 

In order to better prepare current and prospective veterinary team members working in the context of this and future pandemics, we sought to better understand the ethical challenges posed by low and no-contact euthanasia during the early months of the COVID-19 pandemic.

## 2. Materials and Methods

The methodology for this project has been described in detail elsewhere [12]. Briefly, we developed and administered an online, mixed-methods survey to explore the frequency and stressfulness of ethically challenging situations encountered in the early months of the COVID-19 pandemic. The anonymous survey, hosted on the secure web application Research Electronic Data Capture (REDCap), consisted of 29 questions across three sections. Participants were invited to provide free-text responses to three questions: “Since the advent of COVID-19, describe the most COMMON ethically challenging situation you have encountered as a veterinary team member?”; “Since the advent of COVID-19, describe the most STRESSFUL ethically challenging situation you have encountered as a veterinary team member? (If the response is the same as above, enter “same”)”; and “Is there anything else you would like to add about your experience with ethically challenging situations since the advent of COVID-19?” For the first two questions, participants were instructed that the ethically challenging situation identified did not have to be specific to the COVID-19 pandemic. For all questions participants were advised not to include potential identifying information such as names of individuals or workplaces in their responses. In this study, we pooled and analysed the free-text responses to these three questions.

De-identified data were downloaded into Microsoft^®^ Excel for Microsoft Office 365 MSO (16.0.13328.20262). Responses were sorted into categories for the purposes of descriptive statistics. Summary statistics were calculated for the demographic variables using IBM SPSS version 24.

Responses were screened to exclude identifying information, then uploaded onto NVivo^®^ 12 Plus software (QSR International) to facilitate thematic analysis. For this paper, free-text responses referring to the practice of low- and no-contact consultations relating to critically ill patients, or where euthanasia was discussed or performed, were compiled in order to perform a reflexive thematic analysis on this subset of data. Where respondents had written “same” in response to the second free-text question, to indicate that the most common ethically challenging situation was also the most stressful, this second comment was excluded from analysis.

When performed rigorously, qualitative research is explicitly acknowledged to be “context-bound, positioned and situated” [27], with analysis of data reliant on interpretation of the situated researcher. Researcher subjectivity is recognised as a resource rather than a barrier to knowledge production [27]. Thematic analysis is “an *interpretive* activity undertaken by a researcher who is situated in various ways, and who reads data through the lenses of their particular social, cultural, historical, disciplinary, political and ideological positionings” (original emphasis) [28]. It is therefore considered best practice for those performing reflexive thematic analysis to describe their own perspectives, including their “personal and social standpoint, and positioning” [28].

The first author is a companion animal veterinarian, practicing as a primary accession veterinarian and a lecturer in the Sydney School of Veterinary Science. She is also a lifelong companion animal owner. She has been a practicing veterinarian since 2005, well before the COVID-19 pandemic was declared, and has continued to practice since then, modifying her practices in line with public health orders and protocols at practices where she works. Therefore, during the study period, she performed low and no-contact consultations, as well as low-contact euthanasia consultations. The second author is a veterinarian, researcher and lecturer in animal welfare and veterinary ethics at University College, Dublin. She has a long-standing interest in animal welfare science, ethics and law, starting as a student and continuing through practice and into teaching. The third author is a veterinarian, lecturer in epidemiology and public health, and a researcher in the Sydney School of Veterinary Science. His veterinary clinical experience is derived exclusively from government practice as a field veterinarian. He has a strong interest in infectious and transboundary diseases and has conducted original research on the COVID-19 pandemic. Since the declaration of the global pandemic, all authors have engaged either wholly or mostly, in virtual (no-contact) teaching of DVM students. 

Data analysis involved six stages. Firstly, the first author read all comments at least three times. Secondly, initial codes were generated. Each comment was coded inductively for semantic themes, employing a realist approach without a pre-existing theoretical framework. An iterative approach was used. A single comment could be coded multiple times. Where a comment could not be assigned an existing code, a new code was generated. Thirdly, initial themes were generated. Codes were examined to identify clusters of codes and complex codes which were grouped together as themes thought to best represent the data. Themes were reviewed for both internal coherence and distinctiveness from other themes. This involved regularly re-reading all coded extracts from each theme. Where extracts did not fit a theme, these were either reallocated to a more appropriate theme or allocated to a new theme. The fourth and fifth stages—refining themes and developing a thematic map, and defining and naming themes, were performed concurrently, and involved further discussion between all authors. The sixth and final stage involved selection of examples illustrative of each theme. 

## 3. Results

There was a total of 540 valid responses. There were 141 comments, provided by 123 respondents (22.8%). Key demographic frequencies of both the overall respondent population, and the subset who commented on low or no-contact euthanasia in the free-text comments, are summarized in Table 1 and Table 2. Overall, the demographic features of the subset were similar to the overall study population. Briefly, the majority of respondents in this subset were female (*n* = 110; 89.4%) veterinarians (*n* = 98; 79.7%), working in companion animal practice (*n* = 92; 74.8%), and working in Australia (*n* = 69; 56.1%), the USA (*n* = 24, 19.5%), Canada (*n* = 11; 8.9%) and the UK (*n* = 7; 5.7%). Year of birth ranged from 1956–1998, with a mean of 1980 (standard deviation 11.3) and a median of 1982. Year of graduation ranged from 1958–2020, with a mean of 2005 (standard deviation 11.1) and a mean of 2007. 

**Figure 1:**
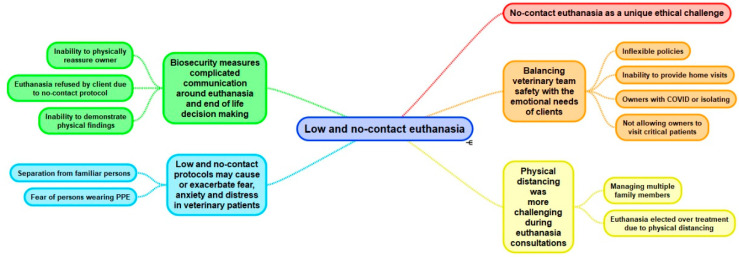
Thematic map depicting themes and subthemes constructed through reflexive thematic analysis.

We identified five major themes relating to euthanasia: no-contact euthanasia as a unique ethical challenge; balancing veterinary team safety with the emotional needs of clients; low and no-contact protocols may cause or exacerbate fear, anxiety and distress in veterinary patients; physical distancing is more challenging during euthanasia consultations; and biosecurity measures complicated communication around euthanasia and end- of-life decision making (Figure 1).

### 3.1. No-Contact Euthanasia as a Unique Ethical Challenge

A number of respondents raised no-contact euthanasia as the most common, the most stressful or both the most common and most stressful ethically challenging situation (ECS) encountered since the beginning of the pandemic, due largely to the absolute exclusion of owners:

“Disallowing witnessing euthanasia”

veterinarian, Singapore

“Putting animals to sleep without owners allowed to be present”

veterinary nurse, UK

“Having to make owners stay outside while we take their pet inside”

veterinary nurse, Australia

The third comment in particular suggests externally imposed rules or protocols followed by veterinary team members that disallow owner presence.

### 3.2. Balancing Veterinary Team Safety with the Emotional Needs of Clients

Some respondents described the challenge of managing the conflict between ensuring the safety of the veterinary team, through strict physical distancing, while meeting the needs of clients to be present during euthanasia of an animal. A veterinary nurse from the USA described the difficulty in weighing up the costs (to the veterinary team) of allowing clients to be present, against the emotional costs (to the client) of not allowing them to be present during euthanasia:

“Being forced to choose between allowing clients into the facility for a euthanasia or maintain “no client access” policies instituted to reduce potential exposure and allow for social distancing. By allowing clients to be present during a euthanasia there is a risk of exposure to both our staff members and the clients in question. It uses scarce and valuable PPE and adds further stress to the team in an already emotionally taxing situation. However, denying clients the opportunity to be present during the euthanasia compounds the grief and loss of an already deeply traumatic situation and denies them a sense of closure and control.”

Veterinary nurse, USA

Other respondents confidently prioritised the safety of the veterinary team, justifying it as “…the ethical decision to protect our staff.” (Veterinarian, USA).

Some were prepared to take a calculated risk in breaching workplace protocols excluding owners from attending euthanasia consultations:

“Not being allowed to have owners present or even visit their pet again prior to euthanasia. I found it to be excessive and unnecessary. While I am worried about COVID just as much as the next person and I want to take precautions, I don’t see why we can’t offer the client to be present outside the building on a bench with masks and long extension set etc. I was reprimanded by management for doing just that.”

Veterinarian, Canada

No-contact euthanasia was experienced for some respondents as an ethical challenge even when the owner was self-isolating or diagnosed with COVID-19. An Australian veterinarian described the most stressful ECS they encountered as “…inability of owner to be present for euthanasia when known COVID positive,” underscoring the view that the costs to the owner of not being present were significant, even in the face of high likelihood of exposure of the veterinary team member(s) to the virus in such situations.

Some respondents mentioned not being able to provide a home euthanasia service, due to factors such as reduced staff numbers and increased workplace biosecurity restrictions, as a source of distress, despite justifying such measures as a means of protecting both veterinary team members and clients:

“…having to decline house calls for elderly clients for both our, and their, protection.”

Veterinarian, Australia

For clients such as the elderly, persons living with disabilities and those without transport, house call consultations may have been their only means of accessing veterinary care. For these clients, loss of the house call service may have equated to loss of access to veterinary care altogether.

Some respondents found that not allowing owners into the clinic or hospital to visit critically unwell or dying patients, or even leave a familiar-scented item to comfort an animal due to concerns about fomite transmission of SARS-CoV-2, particularly challenging:

“It is difficult to receive a patient, specifically one that is critical or in pain, and tell the owner they have to wait in their car and/or are not allowed to come in with their pet. Similarly, when owners want their hospitalized pet to have a blanket or shirt with them, it is hard to tell them no.”

Veterinary nurse, USA

For others, not allowing owners to visit critical or dying animals transgressed their values about acceptable care of animals and their owners:

“Not being able to provide clients contact with their seriously ill hospitalized pet…it is not what I would consider acceptable for my own pets.”

Veterinary nurse, Australia

One respondent referred to a client-free hospital as the “ideal”, but described weighing up the needs of both the veterinary team and the client, suggesting a possibly flexible approach:

“Balancing the needs of clients to see/visit their critically ill pet with the needs of our staff/hospital to maintain a socially distant and ideally client free hospital.”

Veterinarian, Australia

### 3.3. Low and No-Contact Protocols May Cause or Exacerbate Fear, Anxiety and Distress in Veterinary Patients

A number of respondents felt that no-contact euthanasia in particular not only negatively impacted clients, but animal patients themselves, with the key stressor identified as separation from their owner in an unfamiliar environment:

“Many dogs are stressed away from their owner. Also, in the case of very sick animals/emergencies/euthanasias owners are distressed about not being able to be with their animal. Do you cave and let them be there knowing that if you get covid19[sic] the entire clinic team and possibly other clients could get infected, or stick to the policy knowing you are causing emotional distress to the owner and animal?”

Veterinarian, Australia

“Anxious animals being away from their owners creating a more negative environment for the animal to be in.”

Veterinary nurse, Australia

“…distress of pets and owners when separated from [each] other to allow social distancing during exam.”

Veterinarian, Australia

One respondent described being able to implement work-arounds to avoid separation of owners and animals, though did not elaborate on the nature of these:

“People want to be with their animals, and some need to be…sometimes the dog or cat needs them. We finds[sic] ways to accommodate that.”

Veterinarian, Canada

There were concerns that persons wearing PPE may add to fear, anxiety or distress in veterinary patients:

“I thought it was very over the top that in Australia some clinics were either not allowing clients to be present for euthanasia of their pet or required the client to be gowned up in a hazmat suit to be present (and scaring the poor dog with the outfit).”

Veterinarian, Australia

### 3.4. Physical Distancing Is More Challenging during Euthanasia Consultations

Where low-contact euthanasia was performed, respondents described different strategies to maintain physical distancing, including minimising the time in which owners and veterinary team members were in close proximity, performing euthanasia outdoors, the use of intravenous catheters and lines to allow remote injection, and/or minimising the number of people present. One respondent described these extra measures as presenting the most stressful ECS during the pandemic, highlighting the need to separate the client from the animal during the process of euthanasia:

“Not being able to allow clients to be present the whole euthanasia procedure i.e., taking the animal off them in the car park, placing IVC [intravenous catheter], then bringing clients around the back to outside where they must remain for the procedure, never allowing them in the clinic.”

Veterinary nurse, Australia

Some respondents noted concerns about potential increased risk for COVID-19 transmission in euthanasia consultations:

“Being in close proximity to grieving owners (with increased secretions) is stressful on the staff.”

Animal health technician, USA

“Owners crying without masks during euthanasia.”

Veterinarian, USA

Restricting the number of persons present in the euthanasia consultation was the most stressful ECS for some team members, due to concerns about the impact on clients excluded from the procedure:

“…only allowing 1 person to be present when saying goodbye to their pet. It causes moral conflict because it feels wrong asking other family members to leave in a hard time when they are also grieving and would like closure.”

Veterinary nurse, Australia

Some respondents were concerned about the impact of excluding others on individuals forced to attend euthanasia without the support of others:

“Family’s [sic] not allowed to be present during euthanasia. Only one family member outside the building. Seeing the sadness/distress of the one family member shouldering the burden alone.”

Veterinary nurse, Australia

One respondent described physical distancing requirements as a deterrent to work-up of cases for some clients, perhaps leading to premature euthanasia decisions:

“Clients are quicker to elect euthanasia as apposed [sic] to diagnostics as it’s more difficult to bring them into the practice.”

Veterinarian, Canada

### 3.5. Biosecurity Measures Complicated Communication around Euthanasia and End-of-Life Decision Making

Physical distancing complicated communication around euthanasia and end-of-life-decision making. Respondents described the challenge of being unable to demonstrate the clinical status of an animal to the client as they may have done previously:

“Trying to convince an owner that it’s the right time to euthanise their pet when the owner is unable to see their pet’s clinical status and what is happening in the hospital.”

Veterinarian, Canada

In some instances, the prospect of no-contact euthanasia impacted end-of-life decision making. Some respondents reported that the most stressful ECS they encountered was client refusal to euthanise an animal if they could not be present:

“Euthanasia being refused by clients as they cannot be present with their animal.”

Veterinarian, UK

“Clients not wanting to put their pets to sleep as they are unable to attend euthanasia.”

Veterinary nurse, UK

Some respondents noted the lack of contact between themselves and grieving owners as a common or stressful ECS, due to inability to express compassion in a way they were accustomed to:

“It has been difficult to have to refrain from any human touch or closeness during such a personal procedure which requires empathy.”

Veterinary nurse, Australia

“…not hugging the client or spending time with them which we normally do.”

Veterinarian, Australia

## 4. Discussion

Low and no-contact euthanasia of veterinary patients were experienced as stressful by veterinary team members during the COVID-19 pandemic. Traditionally, the veterinary ethical literature has focused on the client, the animal and the veterinarian as key stakeholders [29]. Comments from respondents suggest that veterinary team members were conscious of the needs of a much broader range of stakeholders. Because of the infectious nature of SARS-CoV-2, decisions around whether and how to perform low or no-contact euthanasia also had the potential to impact household members and contacts of clients and veterinary team members, as well as the wider community [12], all with varying risks of viral exposure. Additional stakeholders mentioned included human healthcare workers and the human healthcare system, due to scarcity of resources such as PPE, as well as professional associations, registration boards, charities and non-Government organisations [12]. In addition to complying with professional codes of conduct, veterinary professionals in many jurisdictions were required to comply with public health orders.

The focus of our survey was the frequency and type of ECS encountered during the pandemic and did not attempt to discern the predominant ethical framework(s) utilised by veterinary team members, or whether the ethical approach of veterinary team members shifted with the advent of the COVID-19 pandemic. Nonetheless, responses tended to be most aligned with deontological, utilitarian or virtue ethics approaches. Deontology holds that an action is good or right if it conforms to a rule or a moral norm, and prioritises the intentions of the decision-maker [30]. The theme “*No-contact euthanasia as a unique ethical challenge”* comprised comments about following rules, for example, “disallowing” clients from being present during euthanasia, as well as comments indicating distress about the inflexibility of such rules, and the consequences, particularly for grieving clients. The emphasis on disallowing owners, or “having to” exclude them from the process, suggests that these veterinary team members were following protocols, or felt compelled by circumstances to act, in conflict with their values. While it may be unavoidable due to workplace policies or public health orders, acting in a way that transgresses one’s deeply held moral beliefs causes moral distress [31]. This can impact the welfare of veterinary team members [32].

In human healthcare, bans on visitors of hospitalised patients, particularly those in ICU and those dying from COVID-19, were instituted around the world [33,34]. These caused distress not just to family members of those patients, but also to healthcare workers [35,36]. The idea of dying alone contravenes beliefs about what is considered a “good death” in many cultures [37]. Selman and others note that “a key clinical debate is whether, and how, to facilitate family members and close friends to be present when someone dies in hospital, hospice or care home during a pandemic” [37]. Family members who are not able to visit dying relatives to say goodbye are at higher risk of developing complicated grief and post-traumatic stress-like disorders [35]. Being unable to see a family member right before, during or immediately after death made it hard for some to accept that the person had died [38]. Given the attachment that many owners have to their animals, it is likely that veterinary clients who were unable to be present during euthanasia may be susceptible to similar negative sequelae.

In our study, many respondents appeared to take a utilitarian approach to decision making around owner presence at the time of euthanasia. Broadly speaking, utilitarians seek to achieve the greatest positive consequences (or the least worst) for the greatest number of stakeholders [30]. This is captured in the theme “*Balancing veterinary team safety with the emotional needs of clients*“. Consider the respondent who posed the question about whether one allows a client to be present “knowing that if you get covid19 [sic] the entire clinic team and possibly other clients could get infected”, vs. not allowing the client to be present, leading to distress for both the animal and the client. However, weighing costs and harms did not necessarily yield a satisfactory approach. Utilitarians evaluate decisions according to their consequences—but the respondent could not have predicted with certainty whether they *would* acquire COVID-19, infect other team members and clients, or indeed how severe such infections would be. Nor could they measure with any certainty the degree of harm to the client or the animal. According to a utilitarian framework, steps taken to mitigate or eliminate the risk of harm are ultimately evaluated according to their consequences, which cannot be known until after those steps are taken. In the context of a pandemic, uncertainty is increased. Interestingly, some commentators attributed moral distress among healthcare workers during periods of extreme resource constraint during the pandemic to a shift in the predominant medical ethic toward utilitarianism [36,39]. Some human healthcare workers (such as veterinarians) breached no-contact protocols in order to “minimise the negative psychological effects caused by not being able to say goodbye and possible ongoing complications of mourning” [38].

Increasingly, the professional identity of veterinary team members has come to be centered around primary concern for animal welfare. Indeed, “protecting and promoting animal welfare” is described as the veterinarian’s “raison d’etre” [40], and is embedded in codes of professional conduct for veterinarians, animal health technicians and veterinary nurses [41,42,43,44,45,46]. This focus on animal welfare has been accompanied by a recognition of the potential iatrogenic harms of veterinary care [47], and concerted efforts to minimise fear, anxiety and distress in veterinary patients [48,49,50,51,52]. For example, the European Veterinary Code of Conduct states that “euthanasia must be practiced with as little pain, distress and fear as possible” (1.2, Recommendation 4) [44]. Yet the theme “*Low- and no-contact protocols may cause or exacerbate fear, anxiety and distress in veterinary patients”* suggests that public health considerations (also embedded in professional codes of conduct), came into conflict with this iatrogenic harm minimisation ethos.

A randomized crossover trial of 44 client-owned dogs examined in the consultation room in the presence of their owner, and the common treatment area (“out the back”) without the owner present, reported higher levels of fear, anxiety and stress in more dogs examined in the common treatment area, without their owners [53]. Similarly, a randomized crossover trial of 21 client-owned cats found that separation from owners and examination in the common treatment area were associated with clinically significant increases in perceived stress in cats [54]. These findings suggest that, where possible, examinations and minor procedures should be performed in the consultation room, with the owner present [49]. At the time of euthanasia in particular, it is recommended to keep the client and patient together throughout the euthanasia appointment “to reduce anxiety for both” [21].

While implemented for the safety of veterinary team members and clients, a potential unintended consequence of no-contact consultations is an increased risk of injury. Some respondents highlighted concerns around safety associated with separation of animals from their owners. Anxious and fearful animals are more likely to scratch, bite or otherwise injure veterinary team members, and may be more refractory to sedation [49,50].

Another common approach to ethics is virtue ethics, which prioritises cultivation of morally relevant, persistent character traits such as compassion, honesty, trustworthiness, integrity and discernment [30]. Virtues are linked to one’s role(s), which may vary. For example, a respondent may have roles as a veterinary team member, a parent, a carer, and a community member. Low and no-contact euthanasia may have led to moral distress for veterinary team members because they were unable to perform their role in alignment with their core values (for example, compassion), or because their professional role as a veterinary team member caring for animals and clients came into conflict with their other roles (for example as a family member or carer seeking to protect those they live with). Indeed, we found conflict between the wellbeing of family/household members and professional role was reported to be among the most common (reported by 46.3% of respondents) and most stressful (33.6%) ethically challenging situations encountered by veterinary team members during the early months of the pandemic [12].

One challenge with virtue ethics is how to manage conflict between different virtues. The finding that, for at least some respondents, *physical distancing was more challenging during euthanasia consultations*, may reflect a conflict between the expectation for veterinary team members to be discerning, to follow reasonable public health orders and to minimise biosecurity risk, and the expectation that veterinary team members are compassionate in the face of the grief of clients and their family members. It can be difficult to navigate conflict between different roles and virtues [30].

Euthanasia, in particular, presents a challenge when physical distancing, as it is a time when veterinary team members must be in close physical proximity to an animal to prepare for and perform euthanasia. It is also commonly a time when owners wish to be close to the animal, bringing them into close proximity with veterinary team members. Prior to the pandemic, the presence of multiple family members, friends, support persons and even other animals prior to, during and after euthanasia of animals was common. Extended appointments for euthanasia were routine, and it was common for multiple persons to attend. This may reflect the reality that “euthanasia appointments are as close to a funeral as some clients will have for their pets” [21]. But extended appointments conflicted with advice to minimise duration of client contact. As stated by several respondents, it is not uncommon for clients, and sometimes veterinary team members, to cry during euthanasia consultations. Tears, along with respiratory droplets, are a potential source of SARS-CoV-2 infection [55], as alluded to by some of the respondents.

Few published protocols for low-contact euthanasia were available at the time. In the experience of the first author (AQ), most veterinary teams in Australia developed their own approaches to low-contract euthanasia on an ad hoc basis, or, where possible, referred clients to home euthanasia services. Indeed, the USA-based Companion Animal Euthanasia Training Academy (CAETA) reported an increase in referrals to home euthanasia services during the pandemic, as well as an increased number of outdoor euthanasias [56], as some hospitals sought to avoid admitting clients onto the premises.

Available guidelines focused on minimising contact time between veterinary team members and clients and reducing the risk of fomite transmission. For example, an early edition of “COVID-19: A guide to reopening veterinary medicine in Ontario” recommended the following:

“Euthanasia appointments should be structured so that time in close proximity to the client is minimized. For example, contactless or quick transfer of the patient, distanced escort of an owner to a room, insertion of a catheter in a separate room, keeping personnel distant from the owner until the time of injection, having the owner stand distant or, if they will hold the animal, have personnel wear PPE to protect themselves (mask and eye protection); Documentation of verbal consent rather than requiring signatures; Using contactless electronic payment wherever possible”

[57]

Some continuing education providers shared strategies for performing low-contact euthanasia. For example, CAETA recommended that house call veterinary team members reduce their exposure by reducing overall appointment volume and minimising the number of people present at euthanasia, screening clients ahead of the appointment for signs of illness, explaining the procedure and collect payment over the phone; dispensing pre-visit pharmaceuticals that clients could administer to animals prior to the appointment to promote sedation and anxiolysis, wearing PPE, requesting that clients present wear PPE, ceasing physical contact with clients (avoid handshakes, hugs), encouraging virtual presence at euthanasia, performing euthanasia outdoors where possible, minimising potential fomite transmission by documenting verbal or electronic instead of written consent, reducing handling of animal bodies and using disposable pads rather than towels beneath animals [58,59].

Euthanasia protocols for anxious or aggressive animals are designed to minimise contact between veterinary team members and the conscious patient, often incorporating oral premedication or sedation [60]. Anecdotally, some teams began using these protocols routinely during the pandemic to minimise contact between veterinary team members and clients. For example, where it was safe to do so, some veterinarians utilized a three-step euthanasia process in canine patients involving (a) oral transmucosal application of detomidine hydrochloride gel (an oral transmucosal preparation typically used to sedate and restrain equine patients, but known to cause reversible sedation in dogs [61,62]) by the owner under the direct supervision of the veterinarian; (b) subcutaneous or intramuscular injection of a sedative agent, and (c) placement of an intravenous catheter in a hindlimb, attached to a long extension set to facilitate pentobarbitone sodium injection at a distance from the patient and clients, or intrahepatic injection of pentobarbitone sodium (J. Campbell, personal communication, December 2021). Non-veterinary team members present would be asked to step away from the dog while injections were given or intravenous catheters placed in steps (b) and (c) but could resume physical contact with the animal once veterinary team members moved away from the patient.

### Biosecurity Measures Complicated Communication around Euthanasia and End-of-Life Decision Making

Biosecurity measures, including low- and no-contact consultations, and the use of PPE—in particular, masks—complicated communication between veterinary team members and clients in general [63], so it is not unexpected that they also complicated communication and end-of-life decision making. Communication that might normally occur in the consultation room may have occurred over the phone or via telemedicine, reducing the ability of both veterinary team members and clients to read non-verbal cues [63]. According to Ware and colleagues, briefer appointments and those where the client is separated from the animal can complicate decision making around treatments, monitoring of outcomes and establishing humane endpoints [64]. 

In human healthcare settings, virtual communication presented a challenge for family members of some patients, including difficulty hearing and unreliable WiFi-connection [65], and could be a source of stress for some family members if not managed appropriately [66]. Video calls could be a source of comfort to some family members [66,67,68], though some bereaved family members and friends displayed an ambivalent attitude to the use of devices to facilitate virtual farewells [37]. Telephone communication was associated with a perceived decrease in communication quality, information and support [69]. Masks reduced the ability to read facial expressions, eliminated lip-reading, and may have reduced audibility of verbal communication [65,70,71].

Veterinary team members typically play an important role in supporting pet owners during end-of-life discussions, euthanasia and the immediate aftercare of the animal’s body [72,73,74]. In a study of 2043 dog and cat owners in the USA, more than half reported that the veterinarian was their primary support in relation to pet dying and death [74]. A systematic review of 19 qualitative papers from 17 studies found that when clients reported positive interactions and high levels of support from veterinarians, they were better able to trust and collaborate, felt more reassured, felt better able to grieve and experienced reduced trauma [75]. Discussions around euthanasia, including the sharing of bad news, quality of life assessment, end-of-life decision making, and comforting grieving clients take time [76,77,78], yet the predominant advice given to veterinary team members was to reduce direct contact time with clients. Due to the circumstances of the pandemic, clients may have wished for more time with veterinary team members. It is possible that the human-animal bond intensified due to changes brought about by the pandemic, including spending more time with companion animals due to working from home, or loss of employment [79]. Isolation may have intensified grief over loss of an animal, particularly owners for whom that animal was their only source of comfort or companionship [79]. 

Respondents reported that, in some cases, the prospect of no-contact euthanasia was a reason for clients to refuse euthanasia. This may have led to situations where euthanasia was delayed, or animals suffered a bad death (dysthanasia). Where veterinary team members had no alternative to no-contact euthanasia (for example, the ability to perform low-contact euthanasia or refer to a service provider who could do so), this likely caused moral distress. It is possible that in such situations, veterinarians continued to treat animals despite poor welfare, or what they felt was futility of treatment. Previous studies have reported that situations in which a client wished to continue treatment despite a patient’s poor quality of life are experienced as ethically challenging by veterinarians [22,24].

## 5. Strengths and Limitations

Limitations of the larger study from which the data discussed in this paper have been discussed at length elsewhere [12,13]. For the purposes of the current discussion, a key limitation was the anonymity of the survey, precluding the opportunity to clarify responses, and explore the social, cultural and contextual factors influencing whether respondents experienced low and/or no-contact euthanasia as ethically challenging. Of the subset of respondents who did report experiencing low and/or no-contact euthanasia as an ethical challenge in the early months of the pandemic, we did not have the opportunity to interview them regarding their experiences, what might have helped them in navigating low and/or no-contact euthanasia, and what they learned from the experience. The focus of our study was ethically challenging situations in general, not specifically low and no-contact euthanasia, which may have limited the extent to which respondents elaborated on this particular topic. However, anonymity may have facilitated more open, honest responses, removing social desirability bias.

The voluntary nature of the survey predisposes it to self-selection bias, whereby those with stronger views on ethically challenging situations may have been more likely to respond. It is possible that those who had more negative views about or experiences with low and no-contact euthanasia were more likely to respond to the survey. Alternatively, those distressed by their experiences may have avoided responding due to concerns about recalling distressing ethical challenges.

While every effort was made to distribute the survey globally, responses came from veterinary team members based in 22 countries. Results are biased towards wealthy, Western countries where the majority of veterinary teams work with companion animals [80,81,82,83,84]. This study may not reflect the experiences of veterinary team members working in other countries.

Nonetheless, this study captures the experiences of veterinary team members from multiple countries during the early months of the global pandemic. It provides a snapshot of ethical challenges around low and no-contact euthanasia at a unique time in history. By its design, it does not document the evolution of ethical challenges faced by veterinary team members during the pandemic. Data were collected in the context of a shortage of PPE, prior to the identification of variants including Delta and Omicron, the availability of vaccinations, and the availability of rapid antigen tests, any and all of which have the potential to modify the likelihood of infection and therefore impact the way veterinary team members and clients behave and interact, including in euthanasia consultations.

This study did not capture the experiences of veterinary clients. While our study provides evidence that low and no-contact euthanasia was a source of stress for veterinary team members, other studies show that low and no-contact veterinary consultations in general were anticipated to be or experienced as stressful by animal guardians/owners. In a study of low-income pet guardian’s experiences at private veterinary clinics and hospitals during the pandemic, interviewees highlighted their inability to accompany the animal during the visit as a stressor both for themselves and for the animal [85]. Interviewees also reported challenges communicating with veterinary team members over the phone.

A survey of 2254 pet owners in the US in a similar time period to this study (April to July 2020) reported that 13% of owners had concerns about accessing veterinary care during the pandemic [79]. Such concerns included protocols that precluded pet owners from accompanying animals during appointments, particularly euthanasia appointments.

A qualitative study of Canadian pet owners with (dis)abilities found that for some, their (dis)ability (e.g., sensory, cognitive or motor) posed a barrier to virtual or telephone consultations or commuting to veterinary clinics during the pandemic [86]. A number reported that the inability to accompany their animals into the veterinary hospital led to distress, and reduced their willingness to access veterinary care [86].

Prior to the pandemic, viral posts on social media platforms Twitter and Facebook implored owners to stay in the room when their companion animals were euthanised, otherwise their pet’s final moments may entail “frantically looking around for their owners” [87]. These posts assume that owners have a choice as to whether to be present during euthanasia but may serve to exacerbate owner distress in situations where this choice is removed, such as in a pandemic.

In light of negative experiences and profound psychological harms suffered by bereaved family members, numerous authors emphatically recommend development of protocols, policies and guidelines to preserve of the ability of family members to visit dying loved ones in healthcare settings and/or be present at the time of death during this and future pandemics [33,37,38,65,66,69].

## 6. Recommendations

Our study highlights a strong need to prepare veterinary team members to navigate ethical challenges presented by low and no-contact euthanasia. As the way euthanasia was discussed and ultimately performed was the main source of concern, we believe that providing further information and training on low-contact euthanasia may help veterinary team members preserve the ability of clients to accompany animals during euthanasia should they wish to do so. This would, in turn, enable veterinary team members to perform euthanasia in alignment with their values, thereby reducing moral distress.

Specific guidelines as to how to assess risk, communicate about and perform low- and no-contact euthanasia in different circumstances, including for example pre-visit pharmaceutical and sedation protocols and checklists for preparing clients, could be included in future editions of guidelines such as the American Veterinary Medical Association’s Guidelines for the Euthanasia of Animals [19], and/or updated biosecurity guidelines distributed by veterinary professional organisations [88].

At various stages in the pandemic, and in some cases throughout the pandemic, veterinary teams have been understaffed and under-resourced, with little time to digest and implement extensive guidelines [12,89,90]. Information contained in biosecurity guidelines and protocols needs to be as accessible as possible. There is an opportunity for professional organisations and continuing professional development providers to train veterinary team members in implementing such guidelines. This includes undertaking risk management with regard to euthanasia consultations, and communication and euthanasia techniques for situations where contact with clients must be minimised or eliminated. Low and no-contact euthanasia guidelines and risk assessment tools should be incorporated in hospital emergency plans.

It would be beneficial if accessible information for clients, for example around the wearing of PPE in the euthanasia consultation, could be developed alongside guidelines and protocols for low and no-contact euthanasia. This information may help reduce miscommunication around practical matters, such as instructing clients how to wear PPE during a consultation and explaining expectations around physical distancing. It may also enable clients to better prepare for euthanasia, particularly if shared ahead of the consultation where possible.

Additionally, customisable templates providing contact details of local support services could be made available to veterinary teams to provide to their clients, ensuring that these details are consistently and accurately communicated. 

Veterinary team members performing or assisting in low-contact euthanasia will require a reliable supply of PPE for themselves, and potentially any clients present. Veterinary professional organisations may have a role in helping to secure a continuous supply chain of appropriate PPE.

## 7. Conclusions

The identification of low and no-contact euthanasia as ethical challenges by over one fifth of respondents underscores that it isn’t just the indications for euthanasia, but the practical aspects of how it is performed, that may be ethically challenging and potentially lead to moral distress for veterinary team members. Wherever possible, no-contact euthanasia should be avoided. Veterinary team members should be better prepared and equipped to perform low-contact euthanasia in the context of this and future pandemics.

## Figures and Tables

**Table 1:** Frequency table providing key demographic information for total number of respondents (*n* = 540) and a subset of respondents who made commented on low and no-contact euthanasia (*n* = 123) in a mixed methods survey on ethically challenging situations encountered by veterinarians, animal health technicians and veterinary nurses globally during the early months of the COVID-19 pandemic in 2020.

Demographic Parameter	Category	Number (Overall Responses, *n* = 540)	Number (Subset of Responses, *n* = 123)	Percentage (% Overall Responses)	Percentage (% Subset of Responses)
Gender	Female	434	110	80.4	89.4
	Male	102	12	18.9	9.8
	Other	4	1	0.7	0.8
Role	Veterinarian	423	98	78.3	79.7
	Veterinary nurse	97	21	18.0	17.1
	Animal health technician	11	2	2.0	1.6
	Other animal health professional	9	2	1.7	1.6
Caseload	Companion animal practice clinical	367	92	68.0	74.8
	Mixed animal practice clinical	38	10	7.0	8.1
	Academia/teaching	34	7	6.3	5.7
	Zoo and/or wildlife practice clinical	27	5	5.0	4.1
	Equine practice clinical	13	3	2.4	2.4
	Exotic/unusual pet practice clinical	12	3	2.2	2.4
	Practice management	13	2	2.4	1.6
	Non-government organisation	10	0	1.9	0
	Scientific research/laboratory animals	8	0	1.5	0
	Government	8	0	1.5	0
	Other	5	0	0.9	0
	Industry (e.g., pharmaceutical companies, food companies)	4	1	0.7	0.8
	No longer working as a veterinarian	1	0	0.2	0
Country	Australia	316	69	59.1	56.1
	United States of America	125	24	23.1	19.5
	Canada	26	11	4.8	8.9
	United Kingdom	25	7	4.6	5.7
	New Zealand	12	6	2.2	4.9
	Singapore	10	2	1.9	1.6
	Germany	6	3	1.1	2.4
	China	4	0	0.7	0
	Netherlands	3	1	0.6	0.8
	Other *	13	0	2.4	0

* Other included one respondent (0.2%) from each of the following countries: Austria, Belarus, Cambodia, Denmark, France, Hong Kong, Republic of Ireland, Jamaica, Lithuania, Mexico, Spain, Thailand, Zimbabwe. Percentages may not add to 100 due to rounding to one decimal place.

**Table 2:** Descriptive information for continuous exploratory variables (year of birth, year of graduation) for total number of respondents (*n* = 540) and a subset of respondents who made commented on low and no-contact euthanasia (*n* = 123) in a mixed methods survey on ethically challenging situations encountered by veterinarians, animal health technicians and veterinary nurses globally during the early months of the COVID-19 pandemic in 2020.

Variable	Range	Mean	Median	Standard Deviation
Year of birth for total number of respondents (*n* = 528)	1926–2000	1979	1980	11.9
Year of birth for subset of respondents (*n* = 120)	1956–1998	1980	1982	11.3
Year of graduation for total number of respondents (*n* = 540)	1958–2020	2004	2007	11.9
Year of graduation for subset of respondents (*n* = 123)	1956–1998	2005	2007	11.1

## Data Availability

The data set presented in this article is not available due to privacy and ethical concerns.
